# Structure and dynamics of optically directed self-assembly of nanoparticles

**DOI:** 10.1038/srep23318

**Published:** 2016-03-23

**Authors:** Debjit Roy, Dipankar Mondal, Debabrata Goswami

**Affiliations:** 1 Indian Institute of Technology Kanpur, Uttar Pradesh 208016, India

## Abstract

Self-assembly of nanoparticles leading to the formation of colloidal clusters often serves as the representative analogue for understanding molecular assembly. Unravelling the *in situ* structure and dynamics of such clusters in liquid suspensions is highly challenging. Presently colloidal clusters are first isolated from their generating environment and then their structures are probed by light scattering methods. In order to measure the *in situ* structure and dynamics of colloidal clusters, we have generated them using the high-repetition-rate femtosecond laser pulse optical tweezer. Since the constituent of our dimer, trimer or tetramer clusters are 250 nm radius two-photon resonant fluorophore coated nanospheres under the optical trap, they inherently produce Two-Photon Fluorescence, which undergo intra-nanosphere Fluorescence Energy Transfer. This unique energy transfer signature, in turn, enables us to visualize structures and orientations of these colloidal clusters during the process of their formation and subsequent dynamics in a liquid suspension. We also show that due to shape-birefringence, orientation and structural control of these colloidal clusters are possible as the polarization of the trapping laser is changed from linear to circular. We thus report important progress in sampling the smallest possible aggregates of nanoparticles, dimers, trimers or tetramers, formed early in the self-assembly process.

Colloidal structures result from coagulation or self-assembly of nanoparticles. Study of colloidal clusters play an important role in understanding macromolecular assembly leading to protein aggregation and clustering^1^,^2^. Yet the *in situ* formation dynamics of colloidal cluster remains to be unraveled. Studies performed under isolated conditions, i.e., when the colloids are extracted from their generating environment, show that the most stable conformers for aggregated colloids have body-centered and face-centered cubic packing^3^. Here we present a direct approach to measure the *in situ* formation dynamics of colloidal cluster by using the optical gradient force-field directed assembly of fluorophore coated colloidal nanospheres into clusters that exhibit a ubiquitous type of energy transfer. This energy transfer signature, in turn, enables us to determine the structure of colloidal clusters. Since these colloidal clusters exhibit shape birefringence, we propose that colloidal cluster shapes can be dictated by the polarization of incident laser beam.

Trapping of multiple particles can occur in case of optical trapping in dense environment such as dense colloidal suspensions^4^, or inside cells^5^ due to the optical gradient forces generated from the tightly focused laser beam. As the optical gradient forces pull all the trapped objects towards the trap center, formation of close-packed optically bound clusters happen (shown schematically in Fig. 1). We specifically demonstrate sampling of smallest possible aggregates of nanoparticles, dimers, trimers or tetramers, formed early in the self-assembly process. We generate colloidal clusters consisting up to four dye-coated polystyrene nanospheres, each of radius 250 nm, under optical tweezers, which provide the necessary optical gradient force-field^6^,^7^. Structures of colloidal clusters are typically studied using light scattering methods^8^,^9^,^10^,^11^. By the same token, we also use backscatter signal from the optical trap. However, while all the other light scattering approaches are used for characterizing isolated clusters, our backscatter signal measurement specifically probes the *in situ* self-assembly process.

Furthermore, in our particular approach, since we have fluorophore coated nanospheres, we have the additional advantage of comparing the light scattering results with their two-photon fluorescence (TPF) signal^12^. In fact, we show that there is an inherent decay present in the TPF signal that enables us to determine the *in situ* structures and dynamics of our colloidal clusters more accurately. When the sizes of the trapped clusters become larger than the focal spot size, partial illumination of our fluorophore coated nanospheres occur (Fig. 1). This results in a decay in the observed fluorescence signal, scaling quantitatively in terms of the ratio of the illuminated to the non-illuminated surface of the partially illuminated colloidal nanospheres^12^. Since decay in fluorescence signal is quantitative with respect to the above ratio, the decay time constants give information about the structure of these trapped clusters. When linearly polarized laser is used for trapping, the optically bound cluster aligns itself with laser polarization while with the insertion of ellipticity in laser polarization, optically bound clusters rotates according to the amount of laser ellipticity^13^,^14^. Rotation of these optically bound clusters due to change in laser polarization leads to different structures and/or orientations that are manifested in changes in TPF decay constants. Using this principle, we are able to probe structure and dynamics of colloidal clusters within the colloidal suspension. Thus we demonstrate great progress in colloid (i.e., nanoparticle aggregate) manipulation and extraction of spatial information.

## Formation of Colloidal Clusters

We measure both the backscatter light as well as the TPF signal from the trapped 250 nm radius dye coated transparent polystyrene nanosphere under the influence of a single Ti:Sapphire laser, which can operate both in continuous and mode-locked condition. This has allowed us to use this single laser as the source for both continuous as well as pulsed optical trapping. The focal spot size under our experimental condition is ~700 nm^12^, which indicates that we are in a position to observe trapping of multiple nanospheres. We are able to observe up to a maximum of four such nanospheres being trapped when they were trapped in a sequential fashion. These nanospheres are studied under such buffered condition that they have the least propensity to aggregate, which was independently confirmed through differential light-scattering experiments conducted at several time intervals. Formation of clusters only occur under the optical tweezing conditions as presented here.

**Figure 2:**
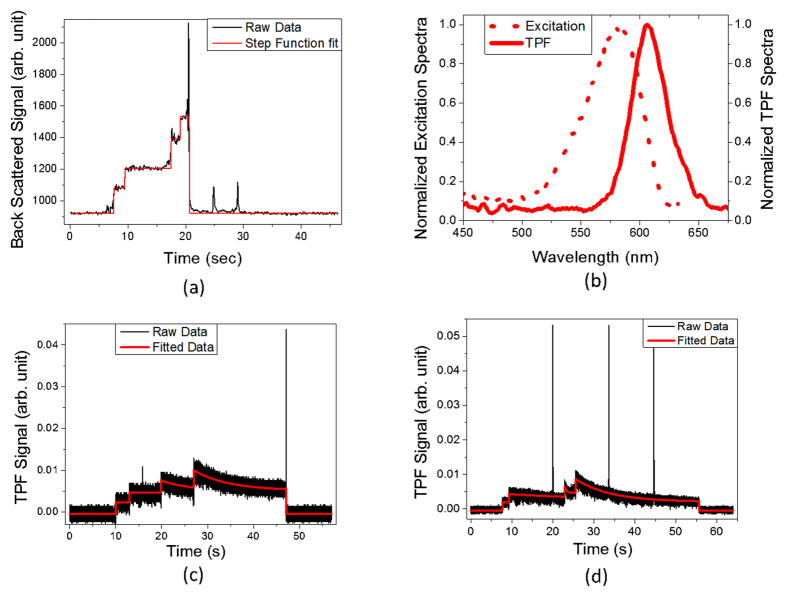
(**a**) Time evolution of backscatter signal from the optically trapped polystyrene nanospheres of 250 nm radius trapped with a femtosecond train of laser pulses. Each step jump in the signal denotes trapping of an additional nanosphere. Nature of the backscatter data is similar for both linear and circular polarized light. (**b**) Single photon excitation and Two Photon Fluorescence spectra of the nanospheres. The broken line corresponds the excitation spectrum and the solid line correspond the TPF spectrum. (**c**) Time evolution of Two Photon Fluorescence (TPF) from optically trapped polystyrene nanospheres of 250 nm radius when trapped using linearly polarized light. (**d**) Time evolution of Two Photon Fluorescence (TPF) from optically trapped polystyrene nanospheres of 250 nm radius when trapped using circularly polarized light. Each step jump in the TPF signal denotes trapping of single nanosphere.

Backscatter light from the trapped nanospheres shows characteristic step-jump signal on silicon photodiode indicative of their self-assembly and aggregation (Fig. 2a). The time evolution of backscatter light from the optically trapped polystyrene nanospheres shows step jumps in backscatter signal. Both the backscatter signal on the photodiode as well as the fluorescence signal on the photomultiplier (PMT) are collected as a function of time. With an increase in the number of trapped objects, the corresponding number of scatterer increases, which, in turn, increases the amount of backscatter signal accordingly in a stepwise manner^7^,^16^. Each step jump in the backscatter signal denotes trapping of an additional nanosphere. The 250 nm radius transparent polystyrene nanosphere surface are also coated with fluorophore molecules that have single photon absorption maxima at ~580 nm and fluorescence emission maxima at ~605 nm (Fig. 2b). When excited with our ~120 fs laser pulses at 780 nm, the fluorophore coated beads show TPF at the focus. TPF signal arises only from the optically trapped nanospheres due to the inherent confocal nature of the TPF imaging technique. As shown in Fig. 2c,d, trapping of a nanosphere shows a sharp rise in the PMT detector signal with respect to background noise indicating TPF. Subsequent addition of each nanosphere into the trapping region causes an initial rise in the TPF signal by an amount corresponding to that of single nanosphere signal^12^,^15^,^16^,^17^,^18^. This initial rise of TPF signal on the PMT correlates well to the photodiode backscatter data and, therefore, independently confirms the backscatter step jump data.

As evident from Fig. 2a, the backscatter signal from the photodiode detector contains a high background value (~920 counts). When the single nanosphere was trapped at ~8 s, the signal increased to ~1080 (signal jump of 160). Trapping of a second nanosphere (at ~9.5 s) would form a dimer, which increases the backscatter signal to ~1200 (signal jump of 120). Such backscatter data can be fitted to a constant step function jump model, where each jump corresponds to an additional nanosphere being trapped (Fig. 2a). Further trapping of third nanosphere occurs at ~17.5 s resulting in the formation of a trimer. This can be seen by the increase in the backscatter signal value to ~1400 (signal jump of 200). At ~19 s, backscatter signal rises to ~1530 (signal jump of 130) due to the further trapping of the fourth nanosphere to form a tetramer. Finally, at ~20.5 s, a large cluster knocks out the tetramer cluster. At ~25 s and ~29 s, trapping of single nanosphere occurs for short times giving rise to spikes in the backscatter signal.

Plot of TPF with time is shown in Fig. 2c for trapping of fluorophore coated polystyrene nanospheres of radius 250 nm under linearly polarized laser light. The sequence of the trapping events showed that the first nanosphere was trapped at ~10 s, followed by the second nanosphere at ~13 s to form a dimer, then the third nanosphere at ~20 s to form a trimer, and finally the fourth nanosphere at ~27 s to form a tetramer. Trapping of each nanosphere led to an increase in TPF signal by ~2.5 mV. All of these trapped nanospheres get out of the trap at ~47 s, which is shown by a sharp decrease in the TPF signal (Fig. 2c).

When trapped with circularly polarized laser light, the plot of TPF with time for trapping of polystyrene nanospheres of radius 250 nm is shown in Fig. 2d. Sequential trapping of four nanospheres occurred at the following time intervals: the first nanosphere at ~8 s, second nanosphere at ~9.3 s to form a dimer, third nanosphere at ~23 s to form a trimer and fourth nanosphere at ~26 s to form a tetramer. A sharp fall of TPF signal to its initial background signal value at ~56 s indicates that the trapped tetramer is getting out of the trap. We could observe up to a maximum of four nanospheres trapped at the same time as was the case of optical trapping of these polystyrene nanospheres using linearly polarized light. Here trapping of each nanosphere corresponds to ~2.1 mV rise in TPF signal. TPF signal generated by circularly polarized light should ideally be ~0.7 times of the TPF signal generated by linearly polarized light^19^, however, in our experiments this ratio is found to be ~0.8. This deviation from expectation may be attributed to the small polarization scrambling resulting from the tight focusing condition set by the high (numerical aperture) NA objective^20^. The large spikes in TPF signal present in Fig. 2c,b denotes the biased diffusion of large sized clusters through the focal region^21^.

## Discussion

**Figure 3:**
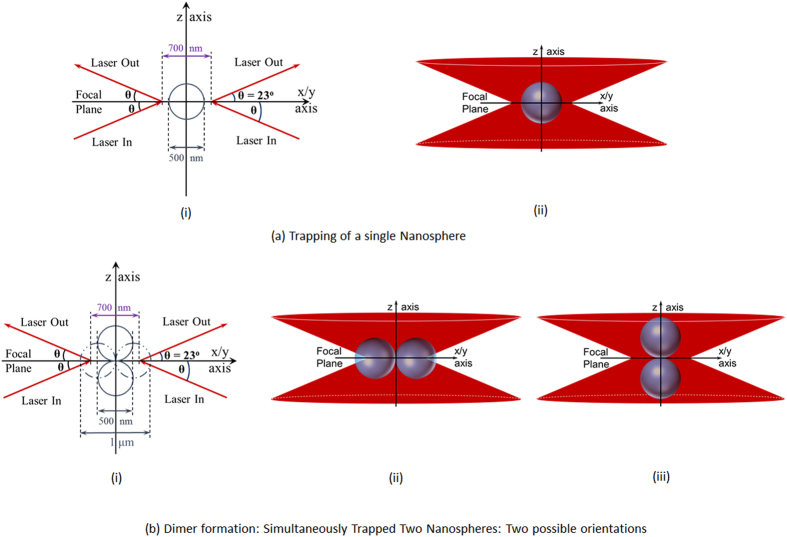
(**a**) Trapping of a single polystyrene nanospheres of 250 nm radius (i) using ray-diagram approach compared to (ii) visualizing the single 250 nm radius nanosphere trapping. The high NA objective produces an angle of incidence, *θ* = 23° into the focal plane and out. (**b**) Trapping of two polystyrene nanospheres of 250 nm radius can result in two possibilities as shown through ray diagram, (i) where one configuration is shown in solid and the other in dashed line. The same is visualized explicitly in (ii) and (iii). For linearly polarized trapping laser, (ii) is less stable as compared to (iii) where the entire bead dimer is contained within the focal volume (700 nm).

When a single nanosphere is trapped, under both linearly and circularly polarized light, TPF signal does not decay with time: instead it fluctuates around its averaged value (Fig. 2c,d). This observation can be explained by the fact that the single nanosphere entirely resides inside the focal region (Fig. 3a), where it is fully illuminated; hence the absence of decay in TPF signal. When a second nanosphere comes in the vicinity of the trap center, due to the strong optical gradient forces, it is also trapped in addition to the existing trapped nanosphere. Due to the strong optical gradient forces, both the nanospheres would prefer to be positioned at the minima of the optical potential well resulting in the formation of an optically bound cluster where the two trapped nanospheres are touching each other. Figure 3b shows the structure and dynamics of the optically bound two-nanosphere cluster, where the laser light travels along the z-axis and the radial axis (r) is in the xy-plane. When trapped under linearly polarized light, the two-nanosphere cluster is aligned with the field propagation direction (z-axis)^22^ as shown in Fig. 3b(i) that was predicted both theoretically^23^ and experimentally^10^. Our present experimental results also confirm this from the fact that in this configuration, no part of the optically bound cluster remains outside the laser illuminated region and hence the TPF signal under linearly polarized light does not decay with time. Since this cluster is dumbbell shaped, it has shape-birefringence, which will make it rotate between (ii) and (iii) orientations of Fig. 3b along the z-axis, due to transfer of spin-angular momentum when trapped under circularly polarized light. Length of this cluster is 2 × (2 × 250 nm) = 1000 nm, which is greater than the focal spot size. Thus when this cluster, due to rotation, will have Fig. 3b(ii) configuration, some portion of it will remain non-illuminated. It can be seen from Fig. 2b that, there is an overlap region between absorption and emission spectra of the fluorophore molecules. Thus, in this configuration, fluorescence energy transfer (FET) process occurs between the fluorophore molecules present in the illuminated surface to those present in the non-illuminated surface, leading to a decay in the TPF signal. In fact, under circularly polarized light, we find a slow counterintuitive single exponential temporal decay in the TPF signal (decay constant, τ ~ 23.5 s). This implies that the optically bound two-nanosphere cluster (dimer) is indeed rotating between (ii) and (iii) configurations of Fig. 3b.

**Figure 4:**
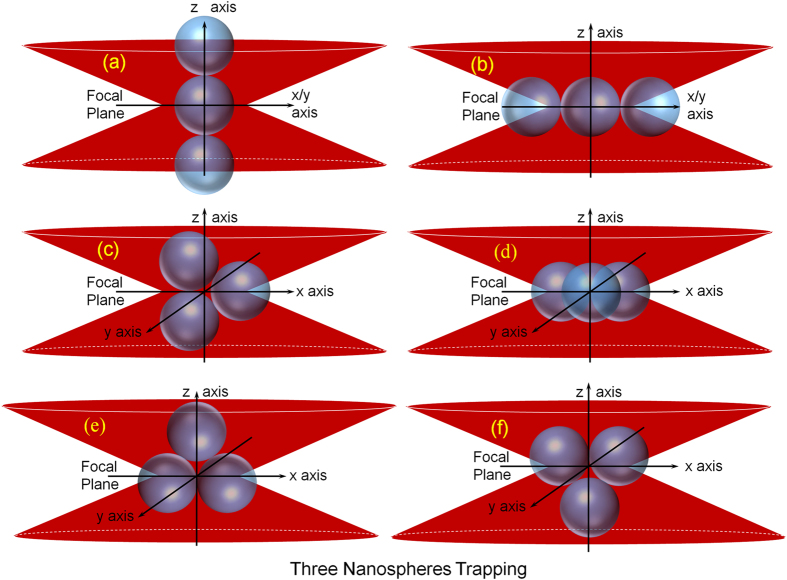
Probable structures and orientations of three 250 nm optically trapped nanosphere cluster (structures (**a**–**f**)).

If a third nanosphere comes close enough to the focal region while two nanospheres are trapped, an optically bound cluster (trimer) consisting three nanospheres can be formed in the trap. Figure 4 shows the possible structures and orientations for this cluster, under the convention that the laser propagation is along the z-direction while the polarization is in the x-y plane. For the linearly polarized laser, polarization is along the x-axis. In analogy to the two-nanosphere case, the expected structure and orientation of the trimer would be configuration (a) of Fig. 4. However, this not the case since the optical gradient force along the axial direction is the least among the three axes^24^,^25^. Due to the predominance of strong optical gradient force, all these trapped nanospheres try to come closer to the focal spot and hence form a three dimensional closed packed structure, which, would be a planer triangular cluster with three nanospheres sitting at the vertices of an equilateral triangle. Thus linear structures (configurations (a) and (b) of Fig. 4) are not possible for this cluster under linearly polarized light.

Considering that the simple harmonic oscillator model for an optical trap is formed by the Gaussian laser beam, it is assumed that the centroid of the three-nanosphere cluster overlaps with the potential energy minimum of the trap center. At the simplest level, it can be assumed that the focal spot is the potential energy minimum of the optical trap. Since the laser is linearly polarized along the x-axis, the gradient force along x, y and z axes are of the order: F_x_ > F_y_ > F_z_^24^,^25^. So two among three nanospheres would be placed along the x-axis and hence (c) and (d) configurations of Fig. 4 are not possible. Similarly, all the other configurations, where two nanospheres are placed along the y-axis, are also not possible. Thus the structure and orientation of this three-nanosphere cluster is either (e) or (f). Among (e) and (f) configurations, one might argue that (e) configuration would be more favored by gravitational forces. However, since the gravitational force is much weaker than the optical gradient forces^26^, we cannot a priori argue for this cluster between (e) and (f), which one is the correct configuration. Both in configurations (e) or (f) of Fig. 4, it can be seen that some portions of the trapped cluster are non-illuminated. This could again lead to the occurrence of FET process between the fluorophore molecules present on the illuminated surface to those present on the non-illuminated surface of the trapped nanospheres due to the Brownian fluctuations of the trapped three-nanosphere cluster even under the linear polarized laser trapping condition. This FET results in an observed exponential decay in the experimental TPF signal with a decay constant of ~5.1 s (Fig. 2c).

The size of the feasible three-nanosphere trapped cluster is always larger than the focal spot size. Consequently, under circularly polarized light, the trapped three-nanosphere cluster will rotate in the xy-plane (i.e., radial plane) due to the spin-angular momentum transfer^5^,^27^,^28^. Experimentally, we find that the FET decay constant of the TPF signal for the three-nanosphere cluster is ~0.5 s (Fig. 2d). This fast decay of TPF signal indicates that, during rotation, the cluster undergoes a change in shape and becomes linear along the radial axis (configuration (b) of Fig. 4) due to the centrifugal force arising from rotation. As TPF decay timescale due to FET shadowing is quantitative with the ratio of the illuminated to the non-illuminated surface of the partially illuminated colloidal nanospheres (M)^12^, we calculate M value for (b) and (e) or (f) configurations of Fig. 4. The FET occurring under such conditions is only of intra-particle nature^12^ and the fully illuminated nanospheres do not contribute to the FET process. The two partially illuminated nanospheres in each configuration are found to be equally partially illuminated. The calculated values of M for (e) and (f) configurations are ~17.93 and that for the (b) configuration is ~2.27. Consequently, the ratio of the M values between configurations (e) and (b) [or (f) and (b)] is ~7.90. This can be compared to the ratio ~10 for the experimental decay constants of the three-nanosphere cluster under linear and circular polarized light. This ~20% deviation in the ratio of M from our model to that of the decay constants from experimental results can be attributed to the fact that the position of the cluster is not exactly at the focal plane as well as the possible increased gap between the trapped nanospheres due to the effect of centrifugal force is not included.

**Figure 5:**
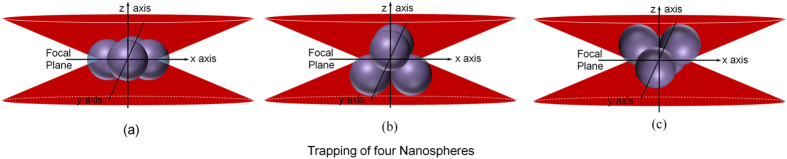
Various possible structures and orientations of four 250 nm nanosphere cluster in optical trap (structures (**a**–**c**)).

When a fourth nanosphere of 250 nm radius comes close to the trap center, while three nanospheres are already trapped, we have found that it is again possible to get the fourth nanosphere trapped due to strong optical gradient force. Continuing with the discussions about the structure of the three-nanosphere cluster (trimer), we can easily conclude that the linear structure for four-nanosphere cluster (tetramer) is also not possible when they are optically trapped under linearly polarized light. The most feasible structures along with their orientations under linearly polarized light are shown in Fig. 5. The trapped tetramer can form either two-dimensional or three-dimensional structures. In case of two-dimensional structures, the most stable and closely packed structure is the rhombic structure formed by the fusion of two equilateral triangles (panel (a) of Fig. 5). Since F_x_ > F_y_ > F_z_^24^,^25^, the short diagonal would be along the x-axis and the long diagonal would be along the y-axis. In case of the three-dimensional structure, the most closely packed structure would be triangular right pyramidal structure (panel (b) and (c) of Fig. 5). Orientation of the three-dimensional cluster would be: two nanospheres placed along the x-axis and the other two nanospheres placed along the y-axis as: F_x_ > F_y_ > F_z_^24^,^25^. Both panel (b) and (c) satisfies this criterion and hence both are equally probable.

All the most probable configurations of four-nanosphere cluster, as shown in Fig. 5, are partially illuminated and thus would show an exponential decay in their TPF versus time plot (which, in fact, can be seen in Fig. 2c). The fact that the decay time of TPF signal is directly proportional to the M value can be utilized effectively to find out, which, among the three panels of Fig. 5, is the actual configuration of this four-nanosphere cluster. Experimentally, the single exponential decay time for the four-nanospheres cluster under linearly polarized light is ~7.3 s. Considering rhombic structure (panel (a) of Fig. 5), we get an M value of ~7.23, while the M value for each of the triangular right pyramidal structures (panel (b) and (c) of Fig. 5) is ~25.93. As the TPF decay time directly corresponds to the M value, the ratio of decay time of four-nanosphere cluster to that of three-nanosphere cluster (trimer) under linearly polarized light (~1.43) should be the same as the ratio of the M values of the corresponding configurations. The ratio of M values for panel (a) of Fig. 5 and panel (e) [or (f)] of Fig. 4 is ~ 0.40, while the ratio of M values for panel (b) [or (c)] of Fig. 5 and panel (e) [or (f)] of Fig. 4 is ~1.45. This comparison clearly points to the fact that the three-dimensional triangular right pyramid structure having two favorable orientations (shown in panel (b) and (c) of Fig. 5) is the correct representation of the four-nanosphere cluster under linearly polarized light. Under circularly polarized light, the TPF decay constant is ~8.4 s. This conveys the fact that, while rotating, the four-nanosphere cluster (tetramer) loosens up due to centrifugal force resulting in an increased M value, which leads to an increase in the experimentally observed TPF decay time value.

Furthermore, these structural understandings can also be justified from our backscatter results: The backscatter signal rise for trapping of a single nanosphere is ~160 counts. For the dimer, the backscatter signal increased by ~120 counts as compared to that of the trapped single nanosphere. This is because the dimer is aligned along the laser beam propagation axis (Fig. 3b(i))^12^,^22^,^23^, and the lower nanosphere somewhat masks the upper nanosphere. Insertion of the third nanosphere into the trapping region to form a trimer results in a rise of the backscatter signal by ~200 counts. This is due to the fact that in the stable orientation of the trimer (Fig. 4e,f), one nanosphere is placed on the center of the laser beam and the two other nanospheres are along x-axis and all the three are touching each other. Finally, when the formation of tetramer occurs from the trimer when another nanosphere is trapped, the backscatter signal rises by a value of ~130 counts. However, it is important to note that the nature of the backscatter signal is similar irrespective of the polarization of the tweezing laser being linear or circular. However, in case of TPF detection, there is distinction between the linear and the circularly polarized trapping laser. Thus, the conjectures for determining structure of the colloidal clusters are better confirmed from TPF decay plots.

Both backscatter and TPF supports the possible orientation of the tetramer cluster predicted from the TPF decay studies as for the tetramer cluster, there is a void space at its center, which lowers the increase of the backscatter signal from the single nanosphere trapping signal rise (~160). But, the backscatter signal cannot distinguish between the rhombic (Fig. 5a) and triangular right pyramidal (Fig. 5b,c) structures, as both these structures contain void space at their center. But TPF decay due to fluorescence energy transfer shadowing technique can easily determine the structure as well as the orientation of the tetramer cluster.

All structures of the colloidal clusters can also be supported, simply from the values of the initial TPF signal for these trapped monomers, dimers, trimers and tetramers as they correspond well to the backscatter signal just discussed. Thus, for trapping with linearly polarized light, the step rise in TPF signal for single nanosphere is 2.28 mV, which with simultaneous trapping of a second nanosphere increases by 2.32 mV. Since both the nanospheres are vertically aligned and fully illuminated, increase in TPF signals are similar. But, when the third particle gets into the trapping region, the initial TPF signal rise of 2.98 mV is comparatively higher. This is due to the fact that, for a trapped trimer, possible configurations (e) and (f) of Fig. 4 are possible, where one nanosphere is fully illuminated while the other two are also maximally illuminated as much as possible geometrically. This leads to a higher increase in TPF signal compared to TPF signal increase from singly and doubly trapped nanospheres. But when the fourth nanosphere is trapped simultaneously, each of the two most possible structures, as shown in configurations (b) and (c) of Fig. 5, have a large void as compared to the possible configurations of the trapped trimer. Thus the initial TPF signal rise of 2.45 mV is lesser than the initial TPF signal of the three nanosphere cluster. The presence of non-illuminated parts as a consequence of geometry constraints leads to decay of the initial TPF signals for trapped trimers and tetramers.

In case of trapping with circularly polarized laser, rise in TPF signal for a trapped single nanosphere is 1.9 mV while that for a trapped dimer, the initial signal rise is 2.39 mV. This can be explained from the fact that during rotation between configurations (ii) and (iii) of Fig. 3b, new portions of second nanosphere gets illuminated, consequently resulting in a higher initial signal rise for the dimer. But, with the simultaneous trapping of the third nanosphere, the configuration as shown in Fig. 4(b) is attained, where only one nanosphere is fully illuminated while the other two are lesser illuminated, which justifies a lesser TPF signal increase of 2.13 mV. Finally, in the case of trapped tetramer, there is a large void due to rotation, which results in an increase in the initial TPF signal of 2.24 mV that is comparable to the trapped trimer case.

In conclusion, we have generated colloidal clusters containing up to four 250 nm radius nanospheres using the strong optical gradient force of a single beam optical trap. Simultaneously we have detected the process of cluster formation *in situ* by using TPF generated from the fluorophore-coated trapped nanospheres. Our approach clearly provides detailed information about multiple trapping events leading to the formation of the optically bound colloidal cluster. We have also studied the structures and orientations of the partially illuminated nanospheres by exploiting the FET shadowing process, which occurs between the fluorophores present on the illuminated surface to those present on the non-illuminated surface. The dynamics of these clusters under circularly polarized light has provided further elaboration of the process. Additionally, change of structure of these clusters under linearly and circularly polarized light has been probed using this method. Thus we demonstrate optical gradient force directed colloidal cluster manipulation and extracted their spatial information and dynamics through FET shadowing.

## Materials and Methods

**Figure 6:**
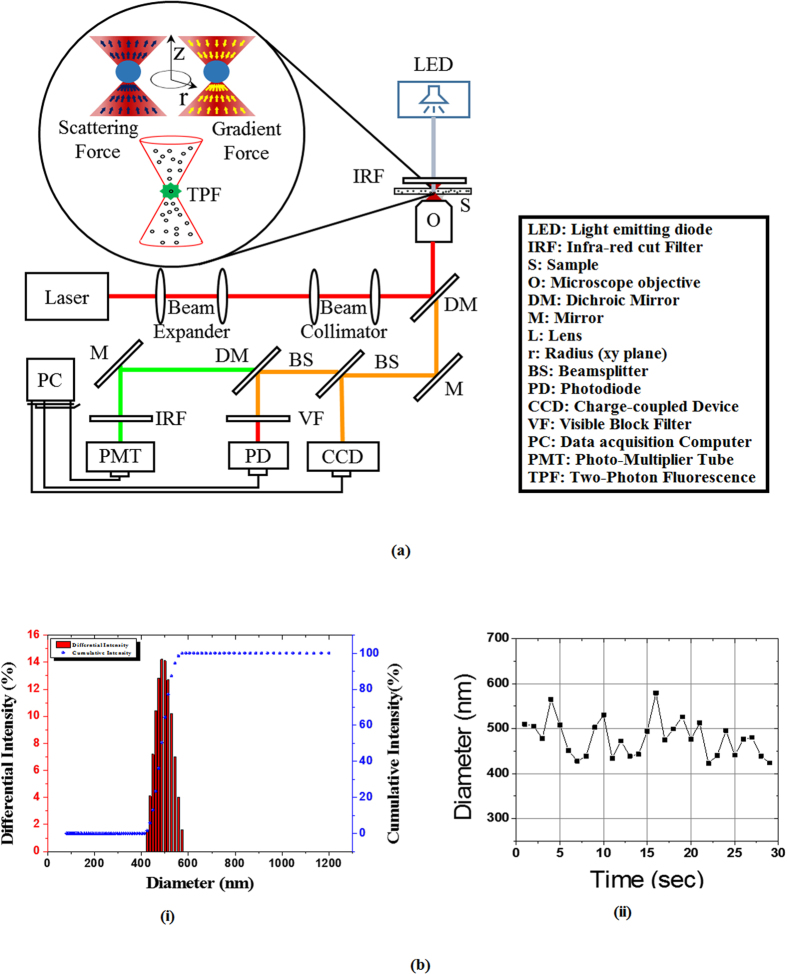
(**a**) Schematic of the Experimental Setup used in our experiments, where the abbreviations indicate the key elements in the setup which is expanded in the legend block. Inset shows the balance of scattering and gradient forces that is critical in the formation of a stable optical trap. (**b**) Measured differential light scattering through our buffered nanosphere sample solution showing (i) the average particle distribution size to be 496 nm and (ii) particle diameter measured at different times.

The experimental setup for our experiments is shown in Fig. 6a. We used a solid-state Ti:Sapphire laser that can operate both in continuous and mode-locked condition (MIRA 900F, Coherent Inc., USA) as the source for optical trapping. To obtain TPF from the trapped objects at low laser power, the laser was operated in the mode-locked condition (pulse-width ~120 fs at 76 MHz repetition rate having central wavelength ~780 nm). The tweezers microscope used for the experiment is a home-built inverted microscope that has been described in detail earlier [12,15, 16, 17, 18]. Most importantly, the trapping laser beam was first expanded and then collimated using telescopic arrangements to overfill the objective back aperture so as to obtain nearly diffraction limited focal spot size. This ensures the maximum tight focusing under the experimental condition. Average power of the trapping laser was kept at 20 mW at the sample stage. To change the laser polarization from linear to circular, a quarter wave plate was placed in the collimated laser beam path. A high numerical aperture (NA) microscopic objective (UPlanSApo, 100XO, 1.4 NA, Olympus Inc., Japan) was used to achieve the tight focusing condition and consequently very high optical gradient force. This same objective also collected the bright field image and the fluorescence from the trapped object. A dichroic mirror that reflects near IR light and transmits visible spectrum of light was placed just below the objective for bright field and TPF images. The trapping event was captured using a CCD camera (350 K pixel, e-Marks Inc.). Due to poor image resolution of the video files, we could not determine the structure, orientations and dynamics of these trapped clusters. As a matter of fact, this is, one of the reasons for us to use two-photon fluorescence as the indicator of occurrence of optical trapping event. The Two Photon Fluorescence signals from the trapped nanospheres were collected using a photo multiplier tube (PMT) (1P28, Hamamatsu) connected to a digital oscilloscope (waveRunner 6100A, LeCroy). TPF spectra of the trapped nanospheres were collected using a multi-mode optical fiber connected to a portable spectrometer (HR2000, Ocean Optics).

Fluorophore coated polystyrene nanospheres of 250 nm radius suspended in water (F-8887, Molecular Probes) were used for trapping. Sample solution was prepared by 100 times dilution of the original stock solution (concentration 2 mM) using Phosphate Buffered Saline solution. Final concentration of our experimental sample is 20 μM. This ensured that the polystyrene beads develop a slight repulsiveness to each other and remain individually in the solution, which was confirmed through differential light scattering performed on our prepared sample solution after storing them at 22 °C for a couple of days (Fig. 6b). All the experiments were performed at 295 K. The temperature change at the focal region due to absorption of laser is <1 K^29^,^30^, which, in turn, has negligible effect (<2%) in the local viscosity of the medium^31^. All the data were analyzed, fitted and plotted using Origin 8.5 (Origin Inc.) software. Only under optical tweezers, formation of clusters occurs. Autodesk Inventor^®^ Professional 2011 (Autodesk Inc.) software was used for analysis of structures and orientations of the colloidal clusters.

## Additional Information

**How to cite this article**: Roy, D. *et al*. Structure and dynamics of optically directed self-assembly of nanoparticles. *Sci. Rep.* **6**, 23318; doi: 10.1038/srep23318 (2016).

## Figures and Tables

**Figure 1:**
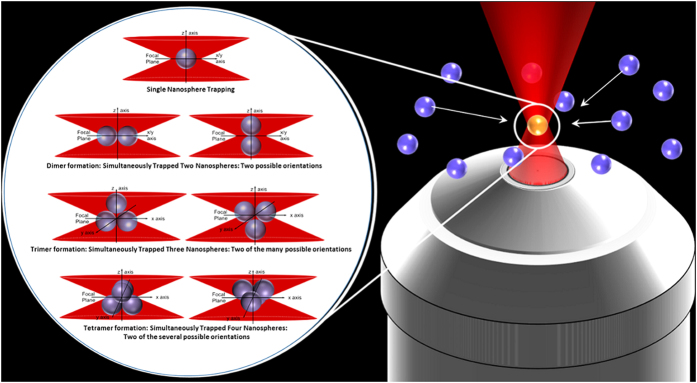
Schematic of the femtosecond optical trap induced early time nanoparticle coagulation process is shown. In case of multiple possible structures, we have chosen to show here two representatives only. Interestingly, we show here, that our approach can provide information on the most probable structures also.
